# Navigator‐assisted transnasal endoscopic approach for optic nerve decompression in a patient with traumatic optic neuropathy

**DOI:** 10.1002/kjm2.12910

**Published:** 2024-12-03

**Authors:** Pin‐Yang Chen, Yi‐Chia Wu, Su‐Shin Lee

**Affiliations:** ^1^ School of Medicine, College of Medicine Kaohsiung Medical University Kaohsiung Taiwan; ^2^ Division of Plastic Surgery, Department of Surgery Kaohsiung Medical University Hospital Kaohsiung Taiwan; ^3^ Regenerative Medicine and Cell Therapy Research Center Kaohsiung Medical University Kaohsiung Taiwan; ^4^ Department of Surgery, Faculty of Medicine College of Medicine, Kaohsiung Medical University Kaohsiung Taiwan

Dear Editor,

Endoscopic trans‐ethmosphenoid optic canal decompression (ETOCD) is a well‐established surgical procedure for traumatic optic neuropathy (TON) that has been demonstrated to improve visual acuity and quality of life.^1^ However, ETOCD is inherently complex due to the intricate anatomy of the operative field, and it necessitates surgeons with specialized endoscopic expertise. Here, we described a novel surgical technique for TON that integrates a combined endoscope and navigation system with preoperative three‐dimensional computer simulation to enhance real‐time surgical accuracy.

A 50‐year‐old man presented with a 4‐week history of visual field defects in his right eye following a road traffic accident. He did not receive any medical intervention prior to this presentation. Physical examination revealed normal findings, with the exception of ptosis of the right eyelid. Ophthalmological examination revealed visual impairment in the right eye, with visual acuity limited to hand motion at 10 cm with perception of light and the remaining visual field restricted to the temporal sector. Additionally, the patient exhibited limitations in extraocular movements (EOM; −4 on abduction, −4 on adduction, −3 on supraduction, and −3 on infraduction). Furthermore, he presented with symptoms consistent with superior orbital fissure syndrome and palsy of cranial nerves 2, 3, 4, and 6. A facial bone computed tomography (CT) scan revealed fractures of the ethmoid bone, with bony fragments impinging upon the right optic nerve (Figure 1A). On the basis of these clinical findings, the patient was given a diagnosis of TON and subsequently underwent surgical intervention by using a navigator‐assisted transnasal endoscopic approach for optic nerve decompression.

**FIGURE 1:**
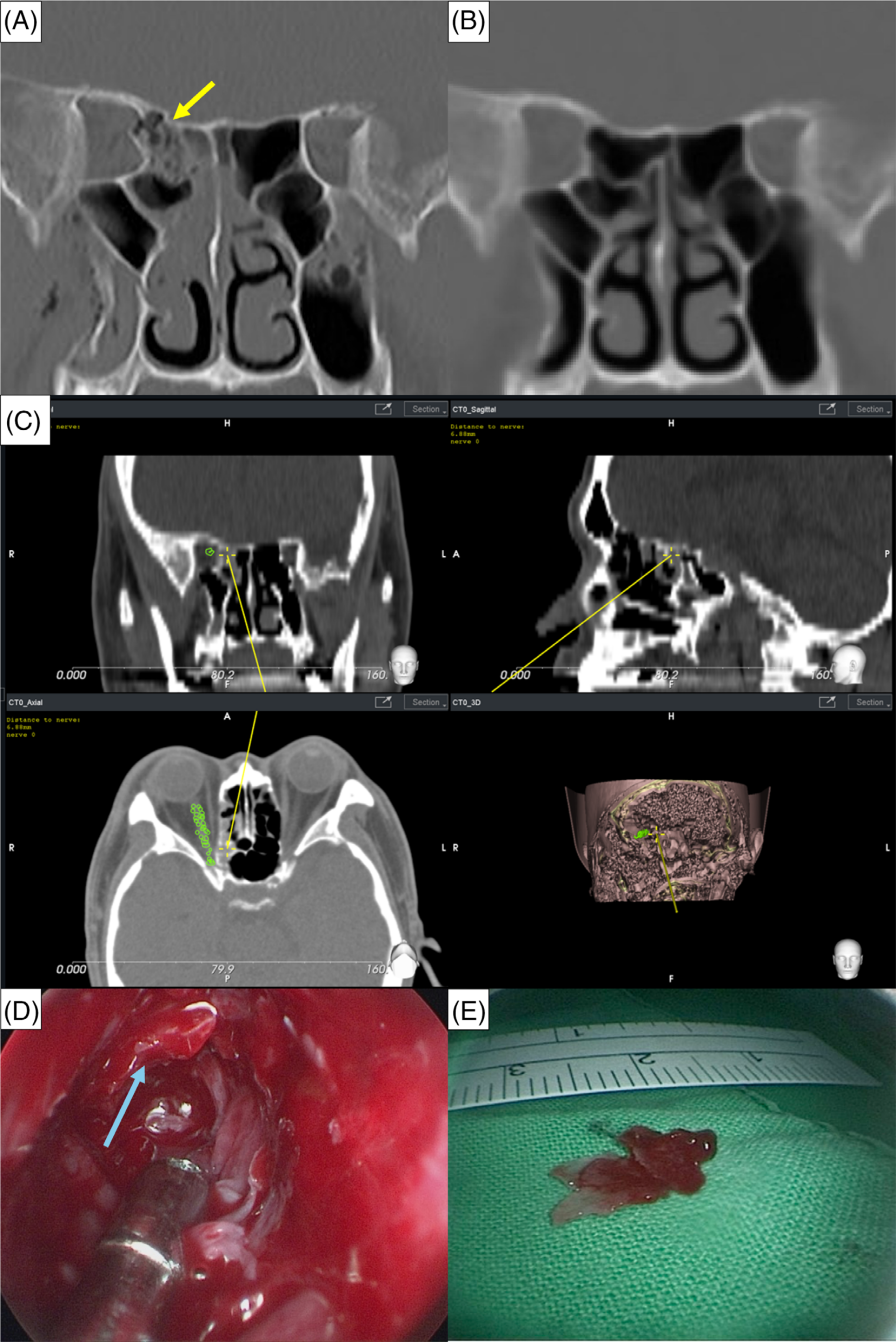
(A) Preoperative coronal CT scan. The yellow arrow indicates bony fragments impinging upon the right optic nerve. (B) Postoperative coronal CT scan. (The left eye served as a reference for lesion location, Figure S1). The scan confirmed successful removal of bony fractures and decompression of the optic nerve. (C) Navigation of instrument tip (yellow cross), marked at a distance of 6.88 mm from the optic nerve (Figure S2). The navigation system is programmed to provide warnings if the tip approaches within 5 mm of the optic nerve, minimizing the risk of iatrogenic injury. (D) Endoscopic view captured from the right nostril depicting ethmoid bone fragments (light blue arrow). (E) This bone fragment seen in 1D was removed, measuring up to 1.3 × 0.7 cm in size.

Preoperative planning for endoscope navigation involved use of the patient's CT data in conjunction with a Retina® navigation system (EPED Corp., Kaohsiung, Taiwan). This virtual planning allowed for precise labeling of the optic nerve in relation to the surrounding bone structures (Figure 1C). A safe margin was established preoperatively, and the system was programmed to provide auditory feedback and warnings if the surgical instruments approached within 5 mm around the nerve.^2^

Under general anesthesia, a transnasal approach to the optic nerve was undertaken, with guidance provided by the navigation system. The endoscope and navigator probe simultaneously passed through the superior nasal concha toward the targeted area. Once in close proximity, the nasal mucosa was carefully retracted using the endoscope to expose the lesion site (Figure 1D). Decompression of the optic nerve was precisely achieved through removal of the ethmoid bone fragments impinging upon the medial orbital wall (Figure 1E).

At 1 month following surgery, the patient exhibited significant improvement in both EOM and visual acuity. Notably, the patient's EOM limitations were reduced to −1 in all directions (abduction, adduction, supraduction, and infraduction). Visual acuity in the right eye improved to hand motion at 200 cm with perception of light. A postoperative CT scan revealed widening of the orbital plate of the ethmoid bone (Figure 1B).

TON is a severe complication of craniofacial injury that often results in substantial vision loss with limited potential for recovery.^3^ Although no consensus has been reached regarding the optimal treatment for TON, optic nerve decompression is considered the cornerstone of surgical management strategies. Various surgical approaches have been developed such as transcranial, transorbital, and ETOCD.^4^ The current report details a novel technique for TON surgery that incorporates a combined endoscope and navigation system. The navigation system offers two distinct advantages. First, the preoperative planning system allows us to mark the tract of optic nerve. This system enables a safe and targeted approach to the nerve with auditory feedback system by precisely separating the mucosa and bony fragments, thereby mitigating the risk of bleeding and cerebrospinal fluid rhinorrhea. Second, the navigation system provides real‐time visualization of the optic nerve, surrounding neurovascular structures, and instrument position, thereby enhancing surgical accuracy.^5^ The present case demonstrates the successful application of this innovative, user‐friendly surgical maneuver in a patient with TON. The combined endoscope and navigation system facilitated a targeted surgical approach, minimizing surgical trauma and achieving successful optic nerve decompression. This technique offers a promising approach for improving surgical outcomes and enables surgeons to feel more confident in performing this surgical procedure.

## CONFLICT OF INTEREST STATEMENT

The authors declare no conflict of interest.

## Supporting information


**Figure S1.** (A) Preoperative coronal CT scan. (B) Postoperative coronal CT scan. The left eye served as a reference for lesion location.

**Figure S2.** Navigation of instrument tip (yellow cross), marked at a distance of 6.88 mm from the optic nerve (green curly line) on Retina® navigation system (EPED Corp., Kaohsiung, Taiwan).
