# Supplementary figures and images for: Navigator‐assisted transnasal endoscopic approach for optic nerve decompression in a patient with traumatic optic neuropathy
Source: Kaohsiung J Med Sci. 2024 Dec 3;40(12):1118–20. doi: 10.1002/kjm2.12910 (PMC11618483; doi:10.1002/kjm2.12910)

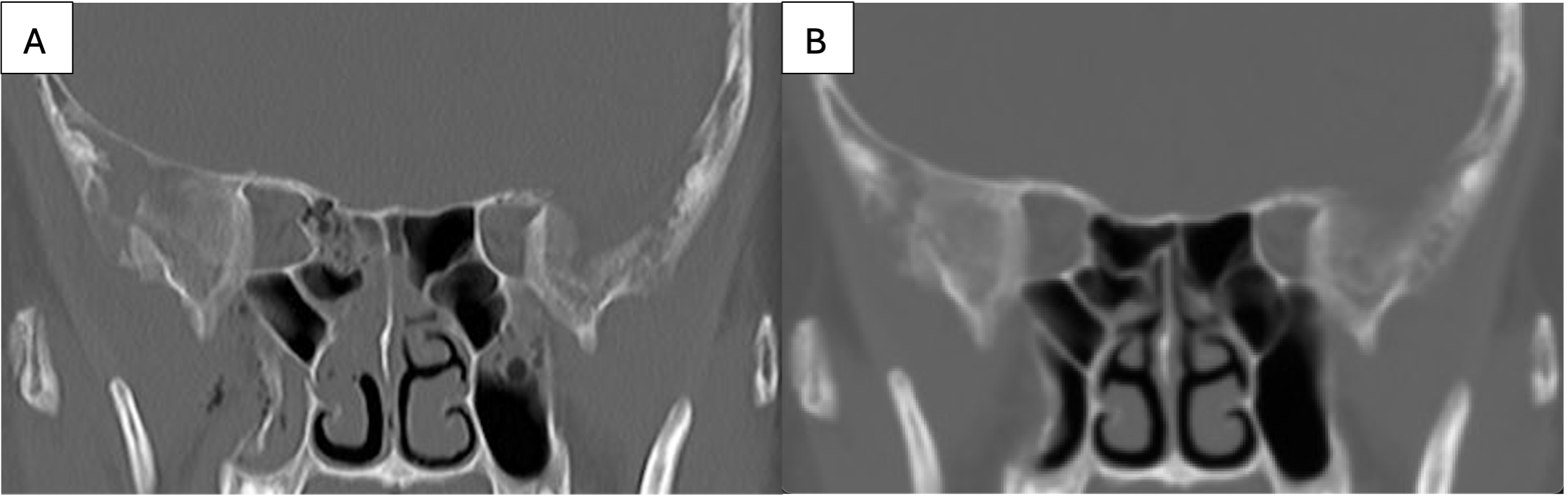

Supplement: Supplementary file 1 — Figure S1. (A) Preoperative coronal CT scan. (B) Postoperative coronal CT scan. The left eye served as a reference for lesion location. [file KJM2-40-1118-s001.tiff]

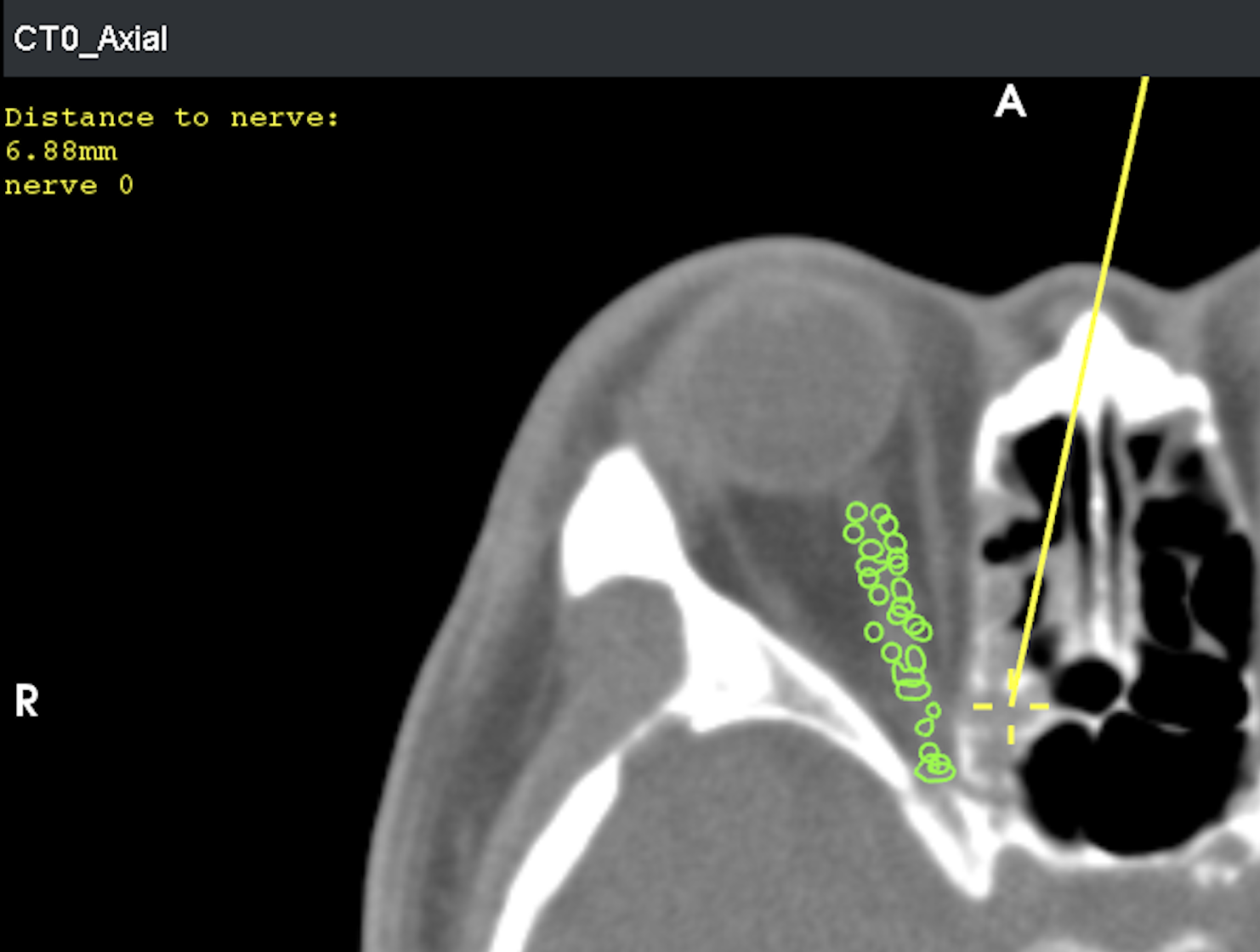

Supplement: Supplementary file 2 — Figure S2. Navigation of instrument tip (yellow cross), marked at a distance of 6.88 mm from the optic nerve (green curly line) on Retina® navigation system (EPED Corp., Kaohsiung, Taiwan). [file KJM2-40-1118-s002.tiff]
